# Vitamin E-Mediated Modulation of Glutamate Receptor Expression in an Oxidative Stress Model of Neural Cells Derived from Embryonic Stem Cell Cultures

**DOI:** 10.1155/2017/6048936

**Published:** 2017-11-20

**Authors:** Afifah Abd Jalil, Huzwah Khaza'ai, Norshariza Nordin, Nur'izzati Mansor, Amirah Salwani Zaulkffali

**Affiliations:** ^1^Department of Biomedical Science, Faculty of Medicine and Health Science, Universiti Putra Malaysia, 43400 Serdang, Selangor, Malaysia; ^2^Genetics and Regenerative Medicine Research Centre, Faculty of Medicine and Health Science, Universiti Putra Malaysia, 43400 Serdang, Selangor, Malaysia; ^3^Department of Nutrition and Dietetics, Faculty of Medicine and Health Science, Universiti Putra Malaysia, 43400 Serdang, Selangor, Malaysia

## Abstract

Glutamate is the primary excitatory neurotransmitter in the central nervous system. Excessive concentrations of glutamate in the brain can be excitotoxic and cause oxidative stress, which is associated with Alzheimer's disease. In the present study, the effects of vitamin E in the form of tocotrienol-rich fraction (TRF) and alpha-tocopherol (*α*-TCP) in modulating the glutamate receptor and neuron injury markers in an* in vitro* model of oxidative stress in neural-derived embryonic stem (ES) cell cultures were elucidated. A transgenic mouse ES cell line (46C) was differentiated into a neural lineage* in vitro* via induction with retinoic acid. These cells were then subjected to oxidative stress with a significantly high concentration of glutamate. Measurement of reactive oxygen species (ROS) was performed after inducing glutamate excitotoxicity, and recovery from this toxicity in response to vitamin E was determined. The gene expression levels of glutamate receptors and neuron-specific enolase were elucidated using real-time PCR. The results reveal that neural cells derived from 46C cells and subjected to oxidative stress exhibit downregulation of NMDA, kainate receptor, and NSE after posttreatment with different concentrations of TRF and *α*-TCP, a sign of neurorecovery. Treatment of either TRF or *α*-TCP reduced the levels of ROS in neural cells subjected to glutamate-induced oxidative stress; these results indicated that vitamin E is a potent antioxidant.

## 1. Introduction

Vitamin E is a fat-soluble compound with antioxidant properties that naturally exists in eight forms (alpha-, beta-, gamma-, and delta-tocopherol and alpha-, beta-, gamma-, and delta-tocotrienol); each isomer possesses unique biological properties [1]. The difference between tocopherol and tocotrienol is the number and position of methyl groups attached to the aromatic ring [2]. In short, tocopherols are saturated forms of vitamin E, whereas tocotrienols possess an isoprenoid side chain. The latter variant of vitamin E only occurs at very low levels in nature, with the highest concentration found in palm oil. Currently, there is increased interest in the tocotrienol-rich fraction (TRF) of palm oil. TRF consists of 25% alpha-tocopherol (*α*-TCP) and 75% tocotrienol. TRF from palm oil has been reported to possess potent antioxidant, anticancer, and cholesterol-lowering activities [2, 3].

At normal concentrations, glutamate functions as a major neurotransmitter in the brain that is critical for cognition, memory, and learning. However, elevated levels of glutamate can cause overstimulation of glutamate receptors, which can excessively excite nerve cells and results in the generation of ROS that can damage cells. Furthermore, glutamate overstimulation was associated with neurodegenerative diseases such as Alzheimer's disease (AD) and Parkinson's disease (PD) [4]. Overstimulation of glutamate receptors such as NMDA, AMPA, and kainate receptors can cause an influx of calcium ions into the postsynaptic membrane. High levels of ATP are required to produce the energy needed to restore the intracellular calcium ion concentrations to normal. This high energy requirement will cause mitochondria to generate more reactive oxygen species (ROS) as a natural byproduct. ROS is a chemically reactive species containing oxygen and includes peroxides, superoxides, hydroxyl radicals, and singlet oxygen [5]. Generally, these molecules are a byproduct of DNA, amino acid, and lipid oxidation that significantly damage cells. Oxidative stress is the condition whereby ROS production is greater than the capacity of the body to reduce oxidation.

This study aims to elucidate the protective role of vitamin E against glutamate toxicity and to understand how vitamin E is involved in modulating glutamate receptor function, antioxidant activity, and neuron-specific enolase (NSE) expression as an injury marker to achieve neurorecovery in an oxidative stress model* in vitro*. Establishment of a transgenic mouse embryonic stem (ES) cell line (46C) provided a method with a renewable source of cells that can generate both neurons and glial cells* in vitro.* This cell line was engineered to express* Sox-1/e*GFP, which is a marker for neural precursor cells and facilitates its differentiation into a neural lineage in an* in vitro* system. In this present study, we mimic oxidative stress in the brain using glutamate excitotoxicity in neural cells derived from the 46C cell line using 4−/4+ protocol as previously described; this protocol successfully generated neural cells* in vitro *to produce a mixed culture of neuronal cells and glial cells [6–8]. It is predicted that both forms of vitamin E (TRF and *α*-TCP) would exert a neuroprotective effect against oxidative stress in neural cultures.

## 2. Materials and Methods

### 2.1. Culture Conditions of Undifferentiated 46C Cells

The transgenic mouse ES cell line (46C) was obtained from Dr. John Orr Mason at the University of Edinburgh, UK. The 46C cells were cultured and passaged regularly on tissue culture flasks coated with a 0.1% gelatine solution. The 46C cell line was cultured in embryonic stem cell medium (ESM), which comprised 1% MEM nonessential amino acids, 1 mM sodium pyruvate, 0.1 mM 2-mercaptoethanol, and 2 mM L-glutamine into 1X Glasgow's MEM (GMEM, Gibco). Complete GMEM media were then aliquoted and supplemented with 15% foetal bovine serum (Gibco) and 10–20 ng/ml human recombinant leukaemic inhibitory factor (LIF) (Merck).

### 2.2. Induction of Differentiation

46C cells were differentiated using 4−/4+ protocols as described by Bain et al., 1995. The 46C cell line was subjected to an 8-day induction procedure which consisted of 4 days of culture as aggregates in the absence of retinoic acid (RA) and 4 days of culture in the presence of RA. To establish the inductions, a confluent culture of undifferentiated 46C cells was dissociated with 0.25% trypsin, and the cell suspension was counted using a haemocytometer. Approximately 5 × 10^6^ cells [9] were seeded in a 100 mm bacteriological grade Petri dish (nontissue culture) in 10 mL of medium (standard medium as mentioned above) without LIF. Cell suspensions were cultured as multicellular aggregates known as embryoid bodies (EBs) for 2 days. The medium was changed, and the cultures were maintained for an additional 2 days. On day 4 of the EBs culture, the medium was changed with the addition of* all-trans-*retinoic acid (Sigma). After another 2 days, the medium was replaced with fresh medium containing RA, and the expression of enhanced green fluorescent protein (*e*GFP) was assessed under an inverted fluorescence microscope (OLYMPUS IX51) to identify neural progenitor cells (NPCs). After an 8-day induction period, the EB suspension was dissociated with a high concentration of trypsin (4X Trypsin-EDTA, 4% chicken serum in 1X PBS) for 5 minutes in a 37°C water bath and agitated to obtain a single-cell suspension, which was then counted using a haemocytometer prior to plating on adhesive substratum dishes precoated with 2 *μ*g/ml laminin from Engelbreth-Holm-Swarm murine sarcoma basement membrane (Sigma) and 10 *μ*g/mL of poly-D-lysine hydrobromide (PDL, Sigma) in 1X PBS. The 24-well plate was coated first with PDL for at least 20 minutes at room temperature, after which the flask was washed with 1X PBS twice before laminin was added and incubated at least 20 minutes at room temperature. Excess laminin was suctioned out before the cells were plated. The cells were seeded at a density of approximately 1 − 2 × 10^5^/cm^2^ for terminal differentiation in serum-free medium comprising a 1 : 1 ratio of DMEM/F12/N2 supplement (Gibco) and Neurobasal/B27 supplement (Gibco). The medium was replaced with fresh N2/B27 complete medium every two days.

### 2.3. Immunocytochemistry (ICC)

The primary antibodies used targeted class III *β*-tubulin (Sigma, T2200) and glial fibrillary acidic protein (GFAP; Abcam, AB10062). Class III *β*-tubulin is a microtubule component that is normally expressed in neuronal cells, and GFAP is expressed in multiple cells in the central nervous system (CNS), including astrocytes and glial cells. On day 6 after neural postplating, the attached neuron-like cells in the 24 well plates were gently washed 3 times with 1X PBS prior to being fixed with 4% paraformaldehyde. The cells were then permeabilized in 1% Triton-X100; blocked with a solution comprising 0.3% BSA, 10% goat serum, and 10% Tween-20 in 1X PBS; and treated with primary antibody. The cells were then incubated with goat anti-rabbit IgG-488 secondary antibodies (Abcam: 150077) for class III *β*-tubulin and goat anti-mouse IgG-488 secondary antibodies (Abcam: 150113) for GFAP, after which the cells were counterstained with 10 *μ*g/mL DAPI (Invitrogen) to detect nuclei. The cells were stored in 1X PBS and kept in the dark until the specimens could be viewed under a fluorescence microscope.

### 2.4. Glutamate Dose Response and Time Course Study

On day 6 after plating the neural cells, the cells in the 24-well plates were challenged with six different concentrations (0, 31.25, 62.5, 125, 250, and 500 mM) of L-glutamic acid monosodium salt hydrate (Sigma) diluted in 1X PBS to calculate the IC_50_ of glutamate toxicity in the cell cultures. The day before glutamate was added, the old medium was replaced with DMEM/F12 and Neurobasal medium (1 : 1 ratio) in the absence of N2 and B27 supplements; this new medium was designated minimal medium. Then, glutamate was administered to the cells and incubated for 24 hours at 37°C in an atmosphere containing 5% CO_2_ and 95% humidity. A similar volume of 1X PBS was added to the cells as a negative control. After 24 hours, 100 *μ*L of MTT (2 mg/ml) was added to each well and incubated with the cells for 4 hours at 37°C in an atmosphere containing 5% CO_2_. Then, 200 *μ*L of DMSO was added to each well to dissolve the formazan crystals. The cells were incubated for an additional 5 minutes in the dark at room temperature and gently agitated for 10–15 seconds, and 100 *μ*L from each well was transferred into a 96-well plate. The absorbance value was measured using an enzyme-linked immunosorbent assay (ELISA) reader (BioTek ELx800, USA) at the 570/630 nm wavelength. The graph of the cell viability against dose and time course was plotted. The IC_50_ and IC_20_ were calculated, and the IC_20_ was used throughout the entire study.

A time course study was conducted on day 6 after neural plating; cells in 24-well plates were challenged with the IC_20_ calculated from the dose response study for 5 different time intervals (0, 4, 8, 12, and 24 hours). After the culture medium was replaced with fresh minimal medium, the calculated IC_20_ of glutamate was added to each well in the plate and incubated for the abovementioned intervals at 37°C in an atmosphere containing 5% CO_2_ and 95% humidity. The cells were then subjected to the MTT protocol as described above.

### 2.5. Posttreatment with Vitamin E on Glutamate-Induced Neural Cells

*α*-tocopherol and TRF were purchased from ICN Biomedical and Sime Darby Malaysia, respectively. TRF used in current study consist of 25% tocopherol and 75% tocotrienols with 95% purity. Vitamin E was dissolved in 100% ethanol and freshly prepared in laminar air flow to maintain its sterility. Working concentration of vitamin E was kept at 0.1% v/v in culture media to avoid toxicity from ethanol. To achieve 0.1% of vitamin E, volume to the total volume of medium, vitamin E was prepared in microgram (*μ*g) concentrations. The vial was wrapped with aluminium foil to protect from light and flushed with nitrogen gas to prevent from oxidation. Vitamin E was then stored at 4°C until further used. To determine the effect of vitamin E as an antioxidant, cell toxicity was induced with IC_20_ of glutamate followed by supplementation of 100, 200, and 300 ng/mL of TRF or *α*-TCP for 24 hours in 5% CO_2_ and 95% air. The cell viability was then assessed using MTT assay. The graph of cell viability against vitamin E treatment was plotted.

### 2.6. ROS Measurement

Cells were seeded in 24-well plates. On day 6 after neural cell plating, the cells were induced with 60 mM of glutamate for 12 hours (based on data from the dose response and time course experiments) followed by treatment with 100, 200, or 300 ng/mL of either TRF or *α*-TCP for an additional 24 hours. A commercially available DCFH-DA dye (OxiSelect™ Intracellular ROS Assay Kit, Cell Biolabs) was used according to the manufacturer's instructions with slight modifications. DCFH-DA diffuses into cells and is deacylated by cellular esterase to the nonfluorescent molecule DCFH, which is rapidly oxidized to the highly fluorescent DCF in the presence of intracellular ROS; this shift in fluorescence is read by a fluorescence microplate reader. The treated cells were washed gently twice with Dulbecco's phosphate-buffered saline (DPBS, Gibco) before 200 *μ*L of DCFH-DA was added to the cells and incubated at 37°C for 1 hour. Then, the cells were washed twice with DPBS and 200 *μ*L of media was added to the cells followed by 200 *μ*L of 2x Cell Lysis Buffer; the mixtures were incubated for 5 minutes. The mixture (150 *μ*L) was then transferred to a black 96-well plate, and the fluorescence was measured using a fluorometric plate reader (FLUOstar® Omega, BMG LABTECH) at 480 nm/530 nm excitation/emission wavelengths. All the data are reported as the mean ± SEM. For the statistical analysis, one-way ANOVA was used; comparisons with the control group were made using Dunnett's test (GraphPad Prism version 5, GraphPad Software). The statistical analysis was presented in Microsoft Excel.

### 2.7. Gene Expression Analysis

To determine the effect of vitamin E as an antioxidant, cell toxicity was induced by 12 hours incubation with 60 mM glutamate, followed by supplementation of 100, 200, or 300 ng/mL of either TRF or *α*-TCP for 24 hours in an atmosphere containing 5% CO_2_ and 95% air. Total RNA was isolated using a FavorPrep™ Blood/Cultured Cell Total RNA Mini Kit (Favorgen). cDNA synthesis was performed using a qPCRBIO cDNA Synthesis kit (PCR Biosystems), and qRT-PCR was performed using qPCRBIO SyGreen Master Mix (PCR Biosystems), following the manufacturer's instructions. The following primer sequences were used:* GluN1*, forward AGTATGACTCCACTCACGG, reverse CATGGTGGTGAAGACACCAGT;* GluK1, *forward CTAATTCGTCTGCAAGAGCTCATC, reverse CTCCTTGCCTTTCTTCATCTCCTT;* NSE*, forward GATCTCTATACTGCCAAAGGTC, reverse GCCTAAGTAACGCTGTTTGTC; and* GAPDH*, forward CAGTATGACTCCACTCACGG, reverse CATGGTGGTGAAGACACCAGT. All the experiments were independently performed at least twice; within each experiment, all the conditions were technically repeated in triplicate. The qPCR data were normalized to the GAPDH values. All data are reported as the mean ± SEM. For statistical analysis, one-way ANOVA was used, and comparisons to positive controls were made using Dunnett's test (GraphPad Prism version 5). Statistical analyses are presented in Microsoft Excel.

## 3. Results

### 3.1. Efficiency of the Neural Differentiation of 46C Cells

**Figure 1:**
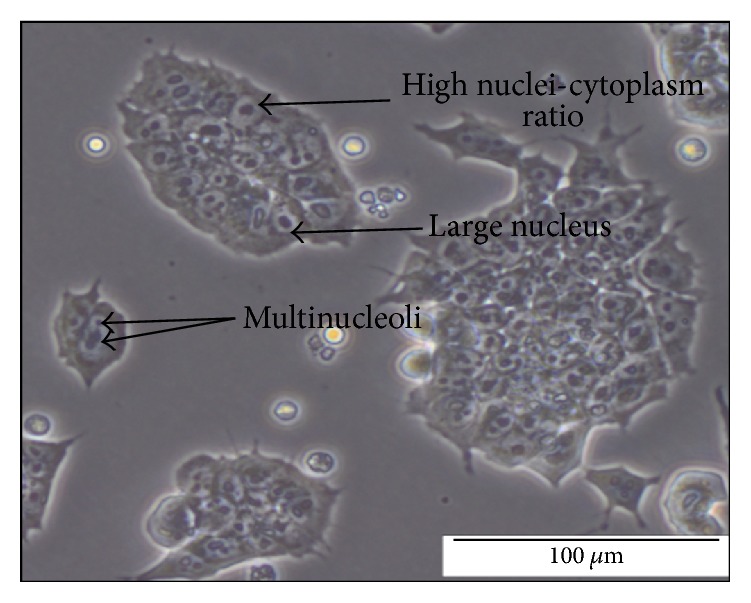
High-quality 46C cells exhibit an elevated nucleus-cytoplasm ratio and large nuclei with multiple nucleoli in culture.

**Figure 2:**
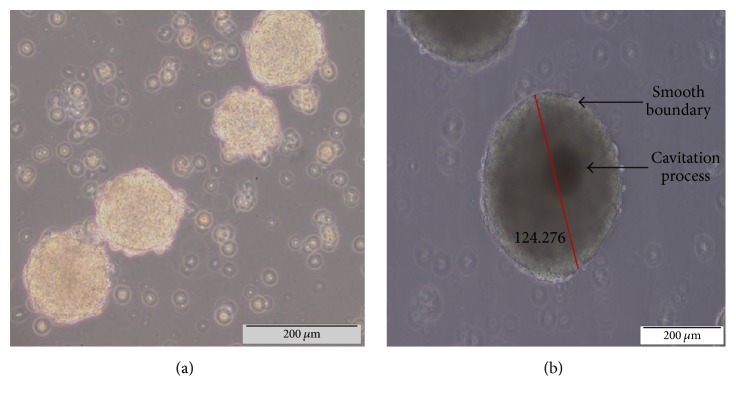
(a) 46C cells begin to form aggregates on day 2 after removal of LIF from the culture and grow on the nonadhesive substratum plate. (b) Mature EBs on day 6 after removal of LIF exhibit a clear and smooth boundary, are larger in size (124.279 *μ*m), and have begun the cavitation process.

**Figure 3:**
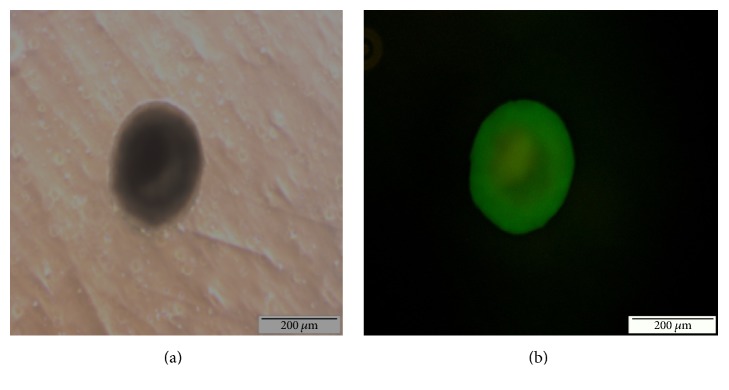
(a) Phase contrast image of day 6 EBs and the corresponding (b) fluorescence microscopy image showing* e*GFP expression, which clearly indicates the expression of* Sox-1* and thus marks the presence of neural precursor cells (NPCs).

In this study, the transgenic mouse ES cell line 46C was used. The quality of the 46C cells was assessed to determine the efficiency of the neural differentiation of 46C cells. High-quality 46C cells exhibit an increased nucleus-cytoplasm ratio and a large nucleus with multiple nucleoli; these characteristics were successfully achieved in this study as shown in Figure 1. Upon removal of LIF and culture of 46C cells on a nonadhesive substratum dish, the cells proceeded to spontaneously differentiate and form EBs. On day 2 after removing LIF, the formation of EBs appeared as small aggregates with an irregular boundary as shown in Figure 2(a). Neural induction was then performed by the addition of 10 *μ*M* all-trans-*retinoic acid (ATRA) from day 4 to day 8 of culturing. On day 6, the EBs appeared mature with clear and smooth boundaries, were larger in size (100–300 *μ*m), and exhibited cavitation within the centre of the masses (Figure 2(b)). From day 5 onwards, the formation of NPCs was observed under a fluorescence microscope for expression of* e*GFP. On day 6, 2 days after the addition of ATRA, cells expressing* e*GFP were detected under a fluorescence microscope as shown in Figure 3(b).

#### 3.1.1. Antigenic Characterization of Class III Beta-Tubulin

**Figure 4:**
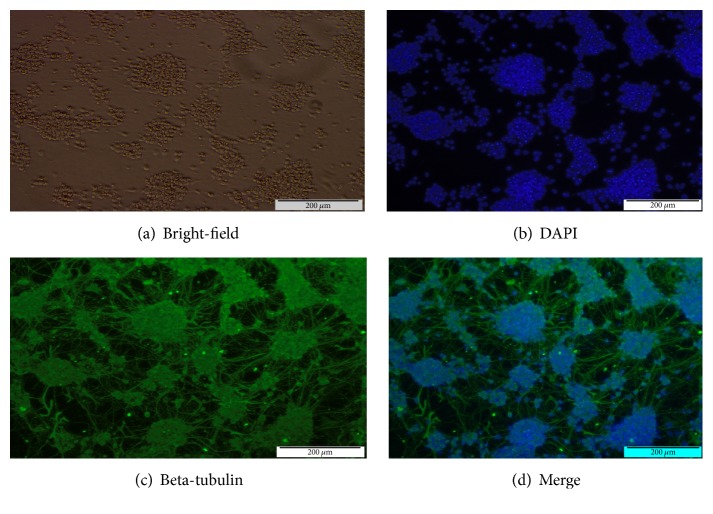
Day 6 of neural postplating. (a) Phase contrast of neural-like cells on day 6 of neural postplating. (b) DAPI counterstaining corresponding to (a). (c) Immunofluorescence of class III *β*-tubulin corresponding to (a). (d) Merge of (b) and (c).

Class III *β*-tubulin is a microtubule component that is expressed in neuronal cells. After the dissociated* e*GFP-positive EBs were replated, the cells began to form a network of neural-like cells after two days. On day 6 after replating, a more prominent network showed neuron-like cells, and class III *β*-tubulin protein expression was assessed via immunocytochemistry. The cells were positive for class III *β*-tubulin protein indicating the presence of postmitotic neurons (Figure 4).

#### 3.1.2. Antigenic Characterization of Glial Fibrillary Acidic Protein (GFAP)

**Figure 5:**
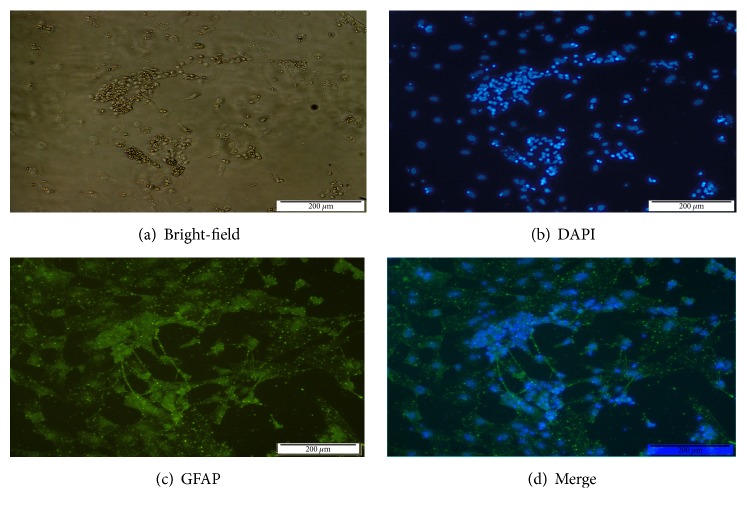
Day 6 of neural postplating. (a) Phase contrast of neural-like cells on day 6. (b) DAPI nuclear counterstaining corresponding to (a). (c) Immunofluorescence of GFAP staining corresponding to (a). (d) Merge of (b) and (c).

GFAP is an intermediate filament (IF) protein that forms a network that provides support and strength to neurons. It is expressed in cells throughout the CNS, including astrocytes and glial cells. On day 6 after plating, the neural-like cells expressed GFAP, which is highly indicative of the presence of glial cells in the cultures of differentiated 46C cells (Figure 5).

### 3.2. Establishment of an* In Vitro* Oxidative Stress Model in a Neural-Derived 46C Cell Line

Glutamate induction was initially conducted in the presence of the N2/B27 supplement; however, the induction failed after many trials, and it was decided that N2/B27 supplementation impeded the glutamate induction. Successful induction was achieved after consistent withdrawal of N2/B27. Glutamate dose response and time course study was then carried out to determine glutamate concentration and time incubation to induced injury in neural-derived 46C cells followed by posttreatment of vitamin E to determine the cell cytotoxicity of vitamin E using MTT assays.

#### 3.2.1. Glutamate Dose Response Study

**Figure 6:**
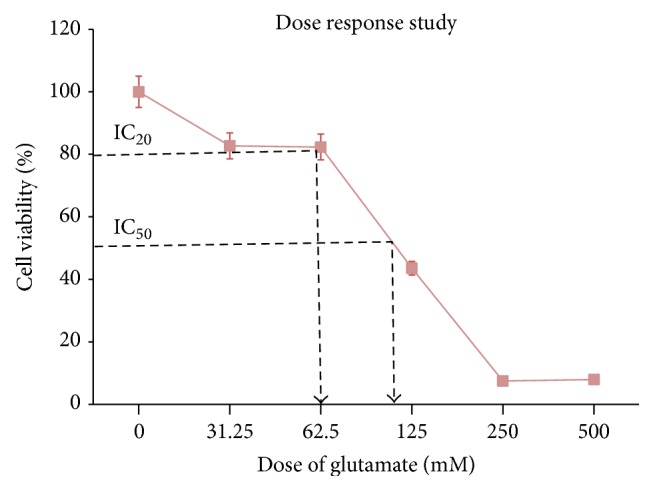
Graph of various glutamate concentrations against cell viability. Cell viability (%) is the mean ± SEM of three independent experiments (*n* = 3 in each experiment).

A dose response curve of glutamate was constructed to determine the tolerance concentration of neural cells derived from 46C cells against glutamate insults (Figure 6). The IC_50_ of glutamate toxicity to induce neural cell was determined; from this value, the IC_20_ was extrapolated and used to induce minimal injury to the neural cells. Figure 6 shows the toxicity of glutamate was dose dependent; with increasing glutamate concentrations, increasing cell death was observed. The IC_50_ and IC_20_ were approximately 125 mM and 60 mM, respectively. Approximately 80% of the neural cells survived when induced with 60 mM glutamate; thus, this dosage was then used for the time course experiment as well as all subsequent experiments.

#### 3.2.2. Glutamate Time Course Study

**Figure 7:**
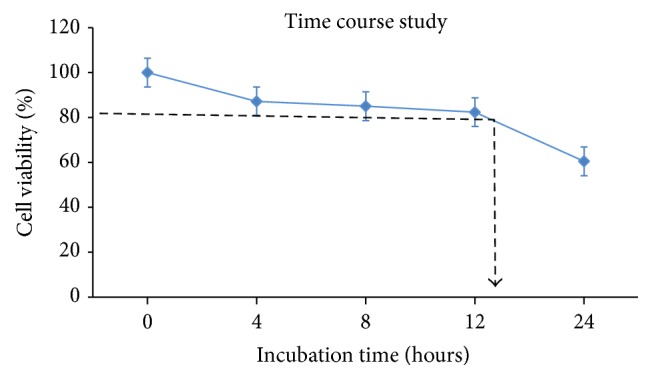
Graph of incubation time against cell viability. Cell viability (%) is the mean ± SEM of three independent experiments (*n* = 3 in each experiment).

Time course study has been conducted in five time intervals: 0, 4, 8, 12, and 24 hours. The purpose of this study is to determine the incubation period of neural cells against glutamate excitotoxicity.  Figure 7 shows incubation time for neural cells to reach 20% cell death with 60 mM glutamate was approximately 12 hours.

From dose response and time course data, neural cells that derived from 46C cells were induced with oxidative stress by 60 mM concentration of glutamate for 12 hours that caused 20% neuronal cell death to generate* in vitro* oxidative stress model. IC_20_ was used to induce minimal injury of the cells; thus prophylactic effects of TRF and *α*-TCP treatment can be determined [10]. IC_20_ was further used in the entire study whereby the cell was designed to only achieve minimal stress. Although the glutamate concentration used to induce neuronal cell injury is high, this concentration is optimal for the setup of this study due to the various sensitivities and resistance to glutamate within the mixed culture.

#### 3.2.3. Effect of Posttreatment with Vitamin E on Glutamate-Induced Neural-Like Cells Derived from 46C Cells

**Figure 8:**
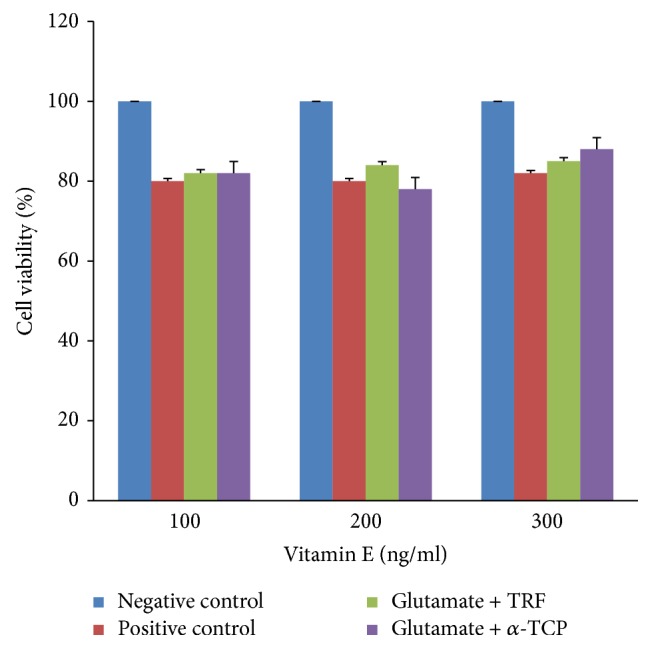
Effects of posttreatment with vitamin E on neural-derived 46C cells injured with 60 mM glutamate for 12 hours before being treated with 100–300 ng/ml of TRF and *α*-TCP for 24 hours (posttreatment). Data are the mean ± SEM of three independent experiments (*n* = 3 per experiment). ^*∗*^*P* < 0.05 compared with negative control; ^Δ^*P* < 0.05 compared with positive control.

The potential of vitamin E in treating the cells after being exposed to high concentration of glutamate was elucidated as in Figure 8. Neural-derived 46C cells were exposed to 60 mM of glutamate for 12 hours. Two different types of vitamin E (TRF and *α*-TCP) of several concentrations (100, 200, and 300 ng/ml) were added to cells. Then the cells were subjected to MTT assay to assess the cell viability.

Twenty percent of cell death occurs in positive control cells upon exposure to 60 mM glutamate. When increased concentrations of TRF were added to the cells from 100 to 300 ng/mL, the cell viability was gradually increased. Nevertheless, this increase was insignificant. Similarly, treatment with *α*-TCP also did not show any significant effects. Treatment with TRF and *α*-TCP (100, 200, and 300 ng/ml) for 24 hours does not give any toxic effect to our neural cell culture. Thus, 100–300 ng/ml of TRF and *α*-TCP were further used to investigate the potential of vitamin E in treating glutamate-injured neural cells.

### 3.3. Effect of Vitamin E on Scavenging Glutamate-Induced ROS in Neural-Like Cells Derived from 46C Cells

**Figure 9:**
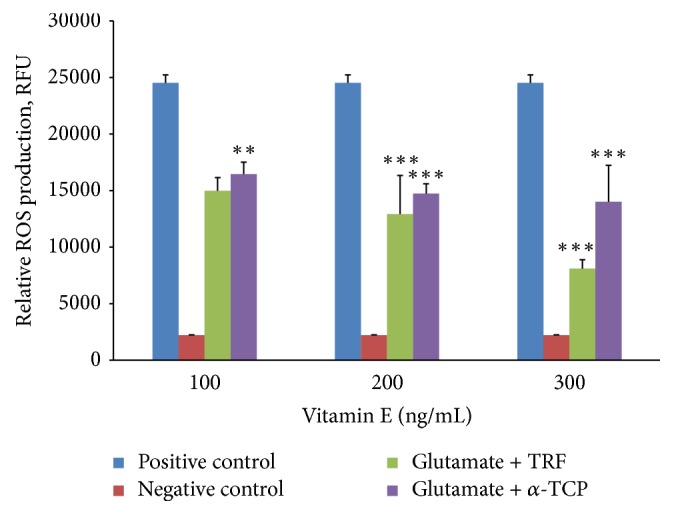
Posttreatment effect of TRF and *α*-TCP on neural-like cells derived from 46C cells injured with 60 mM glutamate. Injured cells (60 mM glutamate for 12 hours) were posttreated with 100–300 ng/mL TRF or *α*-TCP and incubated for another 24 hours. RFU is the relative fluorescence unit where the value is the mean ± SEM of three independent experiments (*n* = 3 per experiment). ^*∗∗*^*P* < 0.01 and ^*∗∗∗*^*P* < 0.001, vitamin E-treated group versus the positive control group.

The posttreatment study was conducted to elucidate the potential of vitamin E to reduce ROS production in glutamate-injured neural cells.  Figure 9 shows that the glutamate insult increased the production of ROS approximately elevenfold in the positive control (Cells + 60 mM glutamate) compared to that in the untreated group (negative control; cell + ethanol).

Regarding the TRF and *α*-TCP treatments, both isomers exerted a similar effect in response to the glutamate toxicity in neural-like cells derived from 46C cells. There were approximately 61.1%, 52.7%, and 33.05% decreases of ROS production upon supplementation of 100–300 ng/mL of TRF, respectively, compared to positive control. Meanwhile, treatment of 100, 200, and 300 ng/mL of *α*-TCP decreased the ROS production by approximately 67.1%, 60%, and 57.2%, respectively, compared to positive control.

ROS production was greatest in the positive control compared to the production in the TRF and *α*-TCP-treated samples. Furthermore, there were significant differences in ROS production among the 100, 200, and 300 ng/mL TRF treatment groups compared with the positive control group. Treatment with *α*-TCP also shows significant differences at concentrations of 200 and 300 ng/mL compared with the positive control. Additionally, TRF exhibited a more marked reduction of ROS levels compared to *α*-TCP.

### 3.4. Effects of Vitamin E on the Gene Expression of Glutamate Receptors and Neuron-Specific Enolase

#### 3.4.1. Glutamate Receptor, NMDA-1 (*GluN1*)

**Figure 10:**
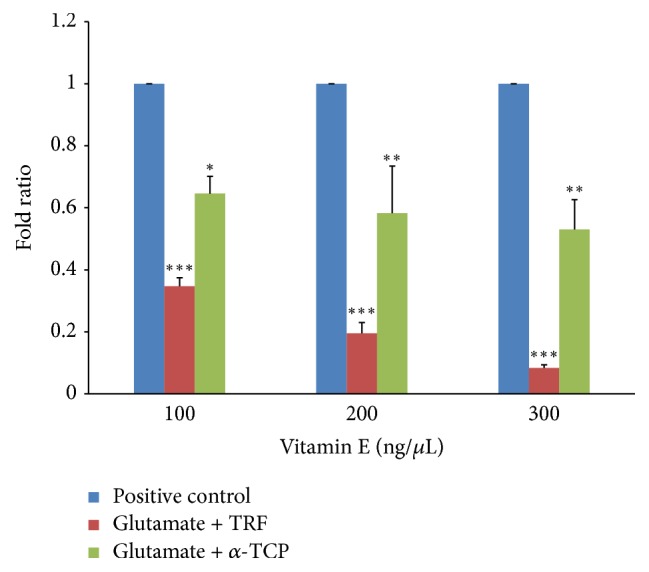
*GluN1* expression in neural cells derived from 46C cells after glutamate challenge and posttreatment with vitamin E. The fold change of* GluN1 *was normalized to* GAPDH* levels. Data are presented as the mean ± SEM of three independent experiments. ^*∗*^*P* < 0.05, ^*∗∗*^*P* < 0.01, and ^*∗∗∗*^*P* < 0.001, vitamin E-treated group versus the positive control group.

In the posttreatment study, 60 mM glutamate increased level of injury as indicated by increased* GluN1 *expression in the neural cells treated with glutamate (Figure 10). However, supplementation of 100, 200, and 300 ng/mL TRF in cells exposed to glutamate toxicity significantly decreased the levels of* GluN1* expression with fold ratios of 0.347 ± 0.03, 0.195 ± 0.04, and 0.083 ± 0.01, respectively. Posttreatment with *α*-TCP also significantly decreased* GluN1 *expression with fold ratios of 0.646 ± 0.06, 0.583 ± 0.1, and 0.530 ± 0.09, respectively. Despite the significantly decreased* GluN1 *expression in the *α*-TCP-treated group, the reduction was only between 40 and 50%. However, the reduction in response to TRF supplementation achieved nearly 90% in the cells treated with 300 ng/mL TRF. When comparing these two isomers of vitamin E, TRF was more effective than *α*-TCP in suppressing* GluN1 *expression.

#### 3.4.2. Glutamate Receptor, Kainate 1 (*GluK1*)

**Figure 11:**
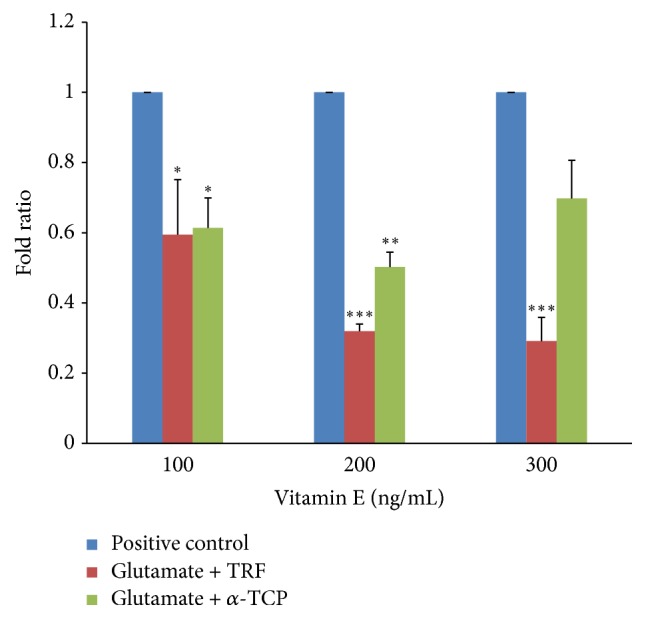
*GluK1 *expression in neural cells derived from 46C cells after glutamate challenge and posttreatment with vitamin E. The fold change of* GluK1 *was normalized to* GAPDH* levels. Data are expressed as the mean ± SEM of three independent experiments. ^*∗*^*P* < 0.05, ^*∗∗*^*P* < 0.01, and ^*∗∗∗*^*P* < 0.001, vitamin E-treated group versus the positive control group.

Figure 11 shows that, upon supplementation with 100–300 ng/mL TRF, there was a significant decline in* GluK1 *expression with fold ratios of 0.594 ± 0.16, 0.320 ± 0.02, and 0.291 ± 0.07, respectively, compared to the positive control. Treatment with *α*-TCP shows a significant difference in* GluK1* expression at 100 and 200 ng/mL with fold ratios 0.614 ± 0.09 and 0.502 ± 0.04, respectively. Overall, 200 ng/mL of either vitamin E isomer elicited the best protection against glutamate insults.

#### 3.4.3. Neuron-Specific Enolase (*NSE*)

**Figure 12:**
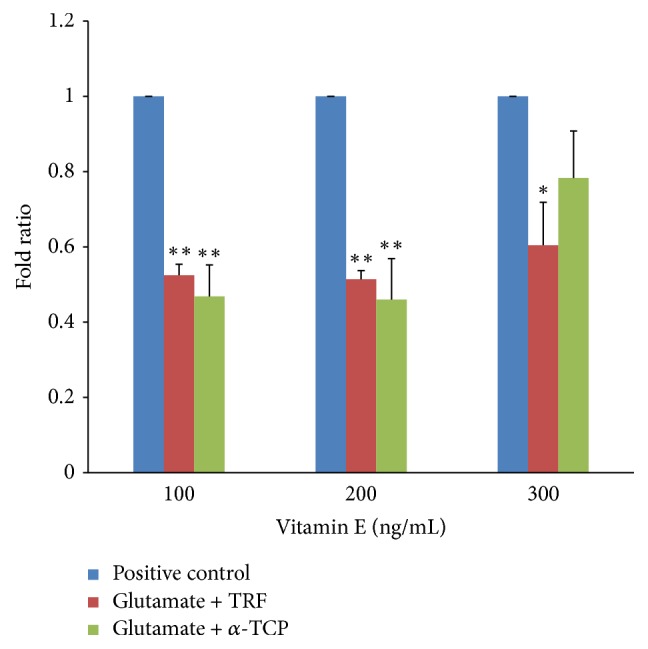
*NSE *expression in neural cells derived from 46C cells after glutamate challenge and posttreatment with vitamin E. The fold change of* NSE *was normalized to* GAPDH* levels. Data are presented as the mean ± SEM of three independent experiments. ^*∗*^*P* < 0.05 and ^*∗∗*^*P* < 0.01, vitamin E-treated group versus the positive control group.

In neural cells derived from 46C cells, treatment with 60 mM glutamate significantly increased the level of injury as detected by the increased levels of* NSE *expression.  Figure 12 shows that, upon supplementation with TRF at 100–300 ng/mL, the* NSE* expression fold ratios were significantly decreased compared to the positive control; these ratios were 0.525 ± 0.03, 0.514 ± 0.02, and 0.605 ± 0.11, respectively. Upon treatment with 100–300 ng/mL *α*-TCP, the* NSE* expression fold ratios in glutamate-injured neural cells were 0.468 ± 0.08, 0.460 ± 0.1, and 0.782 ± 0.12, respectively. A significant reduction of* NSE* expression as an injury marker was achieved by supplementation with 100–200 ng/mL of either TRF or *α*-TCP.

## 4. Discussion

### 4.1. *In Vitro* Oxidative Stress Model

The 46C cell line used in this study is a transgenic mouse embryonic stem (ES) cell that was transduced with the* e*GFP gene inserted into the* Sox-1* open reading frame [11]. In this study,* Sox-1*, which is the earliest marker for NPCs, was used to directly monitor the differentiation of 46C cells under a fluorescence microscope by observing* e*GFP expression in live cells from 46C EBs.* Sox-1* is not expressed in undifferentiated ES cells but it is primarily expressed in NPCs upon differentiation of ES cells into a neural lineage [12]. Our findings show that* e*GFP expression was first detected in 46C EBs on day 6 after neural induction, which indicates the appearance of NPCs in our cultures. A study conducted by Nordin et al. [13] used fluorescence activated cell sorting (FACS) to reveal that that few cells express* Sox1e*GFP before day 4 and that eGFP expression peaks at approximately day 8 in the same cell line.

The present study exhibits the successful differentiation of the 46C cell line into a neural lineage using 4−/4+ protocols with RA as an inducer in the EBs suspension to stimulate neural differentiation. Serum-free media supplemented with N2 and B27 were also used to trigger neural lineages from the 46C cell line. This study revealed the presence of a major population of class III *β*-tubulin-positive cells and a small population of GFAP-positive cells in the cultures. Throughout the experiment, the composition of the cell types after day 6 of neural cell postplating was approximately 80–90% neurons and the remainder were glial cells. The mixed population of neuronal and glial cells in the culture is advantageous because it more closely recapitulates the synergistic effects between glial cells and neurons against neurotoxicity. A study conducted by Kim et al. [14] showed using the same protocol with serum-free medium that a high percentage of class III *β*-tubulin-positive cells concomitant with GFAP-positive cells appeared after 6 days of neural postplating in which the primary population was neurons (approximately 60–70%) and the remainder are glial cells. In addition, Bibel et al. [15] showed that approximately 85% of the cells were class III *β*-tubulin-positive cells with a homogenous appearance of neuronal cell bodies.

In principle, the physiological properties of ES cell-derived neurons are similar to primary neurons with the presence of glutamate receptors and transporters. These neurons can express voltage-gated calcium channels and glutamate receptors such as NMDA and the kainate receptor (David and James, 1999). Neurons derived from ES cells can also use glutamate as a neurotransmitter as reported by Bibel et al. [15], who showed that approximately 93 ± 4.7% of the cells were positive for vGlut1, a vesicular glutamate transporter expressed on the surface membrane of neurons derived from ES cells. Based on this accomplishment, neurons derived from the 46C cell line offer distinct advantages over other neuron-based cell cultures as an* in vitro* model system for investigating nutritional therapy in the prevention or mitigation of AD progression.

The ability of 46C cells to form EBs and express* e*GFP and other neural protein markers denoted the successful implementation and efficiency of the neural differentiation of 46C cells. These neural cell cultures were used as a model of oxidative stress to determine the antioxidant activity of vitamin E on modulating the expression of glutamate receptors and other markers of neuronal injury.

### 4.2. Glutamate Excitotoxicity

In the CNS, glutamate is a major neurotransmitter involved in cognition, memory, and learning [16]. Elevated levels of glutamate can cause glutamate excitotoxicity in neurons and eventually promote a state of oxidative stress. Glutamate excitotoxicity has been shown to contribute to the pathogenesis of neurodegenerative diseases including AD [17, 18]. Oxidative stress occurs due to an imbalance of prooxidant production and antioxidant defence molecules in the body to detoxify the free radicals.

The presence of glial cells (primarily astrocytes) in cell culture can strengthen the neurons and combat the effects of elevated glutamate concentrations. Despite the protection conferred by glial cells against glutamate toxicity, a study conducted by Gupta et al. [19] showed that glutamate toxicity can be induced in a mixed culture of neural cells derived from ES cells by incubating the cells with deprived medium. In parallel, our study revealed that administration of glutamate at a millimolar concentration to cells in the presence of N2 and B27 supplements in the medium failed to induce oxidative stress injury. There are two likely reasons for this response: first, the N2/B27 supplements contain an antioxidant that can neutralize the glutamate toxicity, and second glial cells can provide support against injury. Thus, instead of removing the glial cells from the culture, the N2/B27 supplement was eliminated from the differentiation medium the day before administration of the glutamate treatment to create a nutrient-deficient environment for the cells. Successful induction with IC_20_ of glutamate was then achieved after consistent withdrawal of N2/B27.

In this study, the neural cells were exposed to few concentrations of glutamate and cell viability was assessed to determine the cell response to glutamate excitotoxicity. It was expected that the cell viability would drop significantly when exposed to high concentrations of glutamate. Previous observation found that the exposure of HT4 and HT22 neuronal cells to 10 mM glutamate reduced the cells viability by more than 90% in 24 hours (Sen et al., 2000) [20]. From dose response and time course data, neural cells that derived from 46C cells were induced with oxidative stress by 60 mM concentration of glutamate for 12 hours that caused 20% neuronal cell death to generate* in vitro* oxidative stress model.

To assess the neurorecovery properties of vitamin E against glutamate injury, the posttreatment study was conducted. Various concentrations (100–300 ng/ml) of TRF and *α*-TCP were given to the cells after being exposed to 60 mM glutamate. TRF which consist of 75% tocotrienols and 25% tocopherol were compared with *α*-TCP. The cells viability was later determined by using the MTT assay. Present finding has shown that viability of neural-derived 46C cells was reduced approximately by 20% when exposed to 60 mM glutamate for 12 hours. Posttreatment with TRF increased steadily when the concentrations of TRF given increased from 100 to 300 ng/mL. Although there was no significant difference when compared to the positive control (60 mM glutamate), this result showed that the posttreatment with TRF thus improved cells viability. On the other hand, posttreatment of neural cells with *α*-TCP also produced no significant effect along 100–300 ng/ml concentrations when compared to positive control.

Although the results shown were not significant, TRF exhibited better potential than *α*-TCP which indicates its capability to be used in neurorecovery of injured neural cells. TRF might be able to help the cells recovery after being injured with high concentrations of glutamate. However, MTT assay was mostly used for screening purposes only as it only measures the activity of mitochondrial dehydrogenase in the cells. Therefore, more detailed studies need to be conducted to verify the effects of TRF to reduce the oxidative stress in the neural cells, hence leading to understanding its neuroprotective potential. Thus, in this present study ROS measurement was conducted.

Generally, ROS are formed as a natural byproduct during normal aerobic-based energy metabolism in cells and are safely eradicated by biological antioxidants. Typically, cells have their own protective mechanism against ROS via upregulation of antioxidant molecules such as glutathione, catalase, superoxide dismutase, and glutathione peroxidase to counteract ROS toxicity [21]. The influx of a high concentration of glutamate inhibits the conversion of glutamate and cysteine into glutathione (an antioxidant molecule) through the antiporter system x_c_^−^ [20], which suggests that glutamate excitotoxicity can cause oxidative insults by decreasing the production of antioxidant molecules that can start a chain reaction of free radical attack and eventually result in the disruption of a living cell.

In addition, glutamate excitotoxicity is due to the overstimulation of glutamate receptors—primarily ionotropic glutamate receptors (iGluRs). This current study focused on the gene expression of two iGluRs:* GluN1*, which encodes the NMDA receptor subunit 1, and* GluK1*, which encodes the kainate receptor subunit 1. The results of our experiments indicate that after a 12-hour incubation with 60 mM glutamate, neural cells exhibited markedly increased* GluN1* and* GluK1* expression; excessive stimulation of these receptors mediates calcium ion-dependent cell death [22]. This finding proved that our neural cells derived from 46C cells expressed mRNA for the NMDA and kainate receptors upon incubation with a significant concentration of glutamate. In comparison, according to Gupta et al. [19], an elevated concentration of glutamate can cause overactivation of the NMDA receptor, which leads to cell death in neural cells derived from human embryonic stem cells.

A previous study showed that overactivation of the NMDA and kainate receptors by high concentrations of glutamate caused an excess influx of calcium ions into the cells, which initiates the generation of ROS [4, 5, 23]. Accumulation of calcium ions in the cytosol triggers the depolarization of the mitochondrial permeability transition pore (MTP) that allows the entry of calcium into the mitochondrial membrane and consequently causes the mitochondria to swell and rupture. This action releases the ROS content from the mitochondria into the cytosol and contributes to the high level of ROS in the cytosol during glutamate excitotoxicity.

This study also showed that ROS production in cells injured by excessive glutamate increased approximately elevenfold after a 12-hour incubation compared to untreated cells (negative control). Mouse hippocampal HT22 neuronal cells exhibited more than fivefold increase in ROS production after a 6-hour incubation with 5 mM glutamate [24]. Our finding demonstrates that glutamate is tightly associated with ROS generation in neural cell cultures.

Additionally, another hallmark of neuronal injury is the expression of* NSE*. The current study showed that* NSE* expression was significantly increased in neural cells derived from 46C cells upon glutamate treatment, which indicates successful injury in the culture. According to Martin et al. [25], neurons upregulated* NSE *expression upon neuronal injury due to the high ATP requirements to overcome the injury. During periods of neuronal injury, more glucose is converted to pyruvate via glycolysis to generate more ATP to be utilized by injured cells. This study suggests that the upregulation of* NSE* expression during glutamate insult in this experiment is an effort to promote survival in response to glutamate injury.

### 4.3. Neurorecovery of Vitamin E against Glutamate Excitotoxicity

Vitamin E exhibits strong antioxidant potential that can inhibit the reactivity of ROS or free radicals. Additionally, a few* in vitro* studies reported that vitamin E possesses neuroprotective effects in neurons and astrocytes [2, 10, 26]. In this present study, TRF and *α*-TCP were administered at 100, 200, and 300 ng/mL to determine the ability of these isomers to scavenge ROS and modulate the expression of glutamate receptors and neuron-specific enolase, a marker of neuronal injury.

This present study demonstrates that glutamic excitotoxicity caused neuronal injury via ROS generation. Clearly, vitamin E in the form of TRF and *α*-TCP could reduce the ROS levels in neural cells derived from 46C cells that were subjected to glutamate-induced oxidative stress. Both vitamin E isomers function in a dose-dependent manner and demonstrated a neuroprotective effect due to their strong antioxidant activity. Our data showed that treatment with 100–300 ng/mL vitamin E after a 12-hour induction of glutamate toxicity significantly reduced the generation of ROS, which suggests that the glutamate toxicity causes ROS-mediated cell death and that vitamin E exerted antioxidant potential in neutralizing ROS reactivity.

Furthermore, the current study indicates that supplementation of 100–300 ng/mL of either TRF or *α*-TCP significantly reduced the overexpression of* GluN1 *and* GluK1 *in neural cells subjected to glutamate-induced injury suggesting that vitamin E can potentially modulate glutamate receptor expression and decrease glutamate receptor toxicity. The role of vitamin E in the recovery of glutamate-injured neural cells was also assessed by measuring* NSE *gene expression. After posttreatment with vitamin E, the* NSE* expression levels significantly dropped in neural cells subjected to glutamate toxicity; doses of 100 and 200 ng/mL of either TRF or *α*-TCP exhibited the best neuroprotection against neuronal injury. This result suggests that both TRF and *α*-TCP can improve the survival of injured neural cells at a concentration as low as 100 ng/mL. However, an effective dosage is crucial for vitamin E to exert its beneficial effects, and postinduction administration of 300 ng/mL vitamin E was not as effective as either 100 or 200 ng/mL. When both isomers were compared, TRF consistently conferred better neuroprotection than *α*-TCP in three independent experiments. These results are in agreement with a previous study demonstrating that tocotrienol better protects cells against glutamate-induced injury compared to tocopherol [1, 10].

## 5. Conclusion

The 46C cell line has been successfully used to monitor neural commitments and differentiate into neural cells. Neural differentiation using the single-cell suspension method via the formation of EBs has been shown to efficiently monitor neural differentiation and produce a mixed culture of neurons and glial cells; furthermore, this process has been used to create an oxidative stress model by treating the mixed neural-based cultures with a high concentration of glutamate. The gene expression assay clearly indicated that glutamate receptors (NMDA and kainate receptor) are involved in glutamate excitotoxicity and contribute to oxidative damage of neural cells derived from 46C cells. Alternatively, neural cells have their own protective mechanism towards glutamate toxicity via increased expression of* NSE*, an enzyme critical for glycolysis. The analysis of gene expression revealed that the upregulation of* NSE *is due to increased ATP demand for the neurorecovery process. Upon supplementation with one of two isomers of vitamin E, the ROS levels dropped significantly. Additionally, supplementation with vitamin E suppressed the expression of both tested glutamate receptors as well as* NSE*, which promoted the survival of neural cells derived from 46C cells.

## Abbreviations

AD: Alzheimer's diseases
ATRA: * All-trans*-retinoic acid
EBs: Embryoid bodies
ES: Embryonic stem
ESM: Embryonic stem cell medium
*e*GFP: Enhanced green fluorescence protein
GFAP: Glial fibrillary acidic protein
LIF: Leukaemic inhibitory factor
NPC: Neural progenitor cell
ROS: Reactive oxygen species
RA: Retinoic acid
TRF: Tocotrienol-rich fraction
*α*-TCP: Alpha-tocopherol.
